# Effect of Supervised Resistance Training on Arm Volume, Quality of Life and Physical Perfomance Among Women at High Risk for Breast Cancer-Related Lymphedema: A Study Protocol for a Randomized Controlled Trial (STRONG-B)

**DOI:** 10.3389/fonc.2022.850564

**Published:** 2022-03-01

**Authors:** Karol Ramírez-Parada, Maria Lopez-Garzon, Cesar Sanchez-Rojel, Militza Petric-Guajardo, Margarita Alfaro-Barra, Rodrigo Fernández-Verdejo, Alvaro Reyes-Ponce, Gina Merino-Pereira, Irene Cantarero-Villanueva

**Affiliations:** ^1^ Carrera de Kinesiología, Departamento Ciencias de la Salud, Facultad de Medicina, Pontificia Universidad Católica de Chile, Santiago, Chile; ^2^ 'Cuídate' From Biomedical Group (BIO277), Instituto de Investigación Biosanitaria (ibs.GRANADA), Granada, Spain; ^3^ Department of Physical Therapy, Faculty of Health Sciences, University of Granada, Granada, Spain; ^4^ Unit of Excellence on Exercise and Health (UCEES), University of Granada, Granada, Spain; ^5^ Departamento de Hematología-Oncología, Facultad de Medicina, Pontificia Universidad Católica de Chile, Santiago, Chile; ^6^ Department of Surgery Dr Sótero del Río Hospital, Santiago, Chile; ^7^ Department of Surgery Davila Clinic, Santiago, Chile; ^8^ Centro de Cáncer, Red de Salud U- Christus, Pontificia Universidad Católica de Chile, Santiago, Chile; ^9^ Laboratorio de Fisiología del Ejercicio y Metabolismo (LABFEM), Escuela de Kinesiología, Facultad de Medicina, Universidad Finis Terrae, Santiago, Chile; ^10^ Escuela de Kinesiología, Facultad de Ciencias de la Rehabilitación, Universidad Andrés Bello, Viña del Mar, Chile; ^11^ Departamento Manejo Integral del Cáncer y Otros Tumores, Subsecretaria de Salud Pública, Ministerio de Salud de Chile, Santiago, Chile; ^12^ Escuela de Medicina, Facultad de Ciencias, Universidad Mayor, Santiago, Chile; ^13^ Instituto de Investigación y Postgrado, Facultad de Ciencias de la Salud, Universidad Central de Chile, Santiago, Chile; ^14^ Sport and Health Research Center (iMUDS), Granada, Spain

**Keywords:** breast cancer lymphedema, breast neoplasms, physical therapy specialty, quality of life, resistance training

## Abstract

**Objectives:**

To determine the preventive effects of supervised resistance training on arms volume, quality of life, physical performance, and handgrip strength in Chilean women at high risk for breast cancer-related lymphedema (BCRL) undergoing chemotherapy.

**Design:**

Randomized control trial.

**Participants:**

One hundred and six women at high risk for breast cancer-related lymphedema aged 18 to 70 years.

**Interventions:**

Participants will be randomized into two groups: [a] intervention, who will receive 12 weeks of supervised resistance training (STRONG-B) during adjuvant chemotherapy; and [b] control, who will receive education to promote lymphatic and venous return, maintain range of motion, and promote physical activity.

**Main Outcome Measures:**

The primary outcome will be arms volume measured with an optoelectric device (perometer NT1000). Secondary outcomes will be quality of life, handgrip strength, and physical performance. Primary and secondary outcomes will be measured at baseline, just after the intervention, and 3 and 6 months after. Statistical analysis will be performed following intention-to-treat and per-protocol approaches. The treatment effect will be calculated using linear mixed models.

**Discussion:**

The STRONG-B will be a tailored supervised resistance training that attempts to prevent or mitigate BCRL in a population that, due to both intrinsic and extrinsic factors, will commonly suffer from BCRL.

**Clinical Trial Registration:**

[https://clinicaltrials.gov/ct2/show/NCT04821609], identifier NCT04821609.

## Introduction

Breast cancer is the leading cause of cancer mortality among women worldwide (1–3). While early detection and better treatment strategies have improved overall survival (4, 5), patients often develop adverse effects such as fatigue, pain, sensory loss, impairments in shoulder range of motion and muscle strength, axillary web syndrome, and lymphedema (6–10). These effects ultimately impair survivors’ quality of life and physical performance (11–14).

Among the adverse effects, lymphedema deserves attention because of its chronic nature (15). One-third of breast cancer survivors develop breast cancer-related arm lymphedema (BCRL), 80% of them closely after treatments (16–18). BCRL is an excess accumulation of protein-rich fluid that would otherwise drain *via* the lymphatic system, leading to regional swelling in one or both arms after breast cancer (19, 20). BCRL has multifactorial causes, influenced by treatment strategies and the patient’s ability to form collateral lymphatic pathways post-injury (20, 21). Major risk factors for BCRL are obesity (body mass index ≥30 kg/m^2^), extensive breast and axillary lymph node surgery, radiotherapy in lymph node basin, and taxane chemotherapy (10, 14, 20, 22). Clinically, BCRL is characterized by increased arm volume associated with pain, heaviness, tightness, and a decreased range of motion, thus impacting the quality of life (23, 24).

The treatment for BCRL focuses on volume control through physiotherapy and compression garments (25–27), which represents a high economic burden for patients and the health care system (28). Identifying strategies to prevent BCRL in individuals at high risk, and to improve quality of life of patients is necessary (27).

Historically, there has been objection to promoting physical exercise or weight lifting to breast cancer survivors (29). Nevertheless, this is currently regarded as safe (30). Indeed, there has been an emerging interest in physical exercise (aerobic and resistance) as a safe and effective complementary intervention to prevent or diminish the adverse effects related to breast cancer treatments (31–33).

Exercise increases cardiac output and arterial blood pressure, thus promoting capillary filtration and the entry of fluid and proteins into lymphatic capillaries (34). Exercise also increases lymph propulsion through lymphatic vessels through extrinsic and intrinsic mechanisms, e.g. skeletal muscle pump, respiratory pump, and the pulse of blood vessels near to lymphatic system (34–36). Indeed, lymphatic clearance rates during the initial 15 min of exercise are 5-fold higher than at rest, and remain elevated during exercise (~2.5-fold) (37). Further, physical activity has been shown to improve the quality of life in women with breast cancer (38–40). As mentioned above, physical activity has been also used safely to treat BCRL in survivors (33).

Recent systematic reviews suggest that resistance exercise is a potentially effective strategy to prevent BCRL in women (30, 41); however, few studies included women exclusively at high risk of BCRL (33, 42). The results reported may thus not apply to women at high risk of BCRL. In addition, the studies including women at high risk of BCRL used resistance training with light loads (33). Light training load has a small effect on muscle strength and morphological adaptations, and minor improvements in physical performance and quality of life (41). In contrast, acute (43) and cumulative (42) heavy-load resistance exercises improved the quality of life, muscle strength, and physical performance without increasing risk for BCRL.

Therefore, this study will aim to determine the preventive effects of supervised resistance training on arms volume, quality of life, physical performance, and handgrip strength in women undergoing adjuvant chemotherapy with a high risk of BCRL.

## Methods

### Study Design

**Figure 1:**
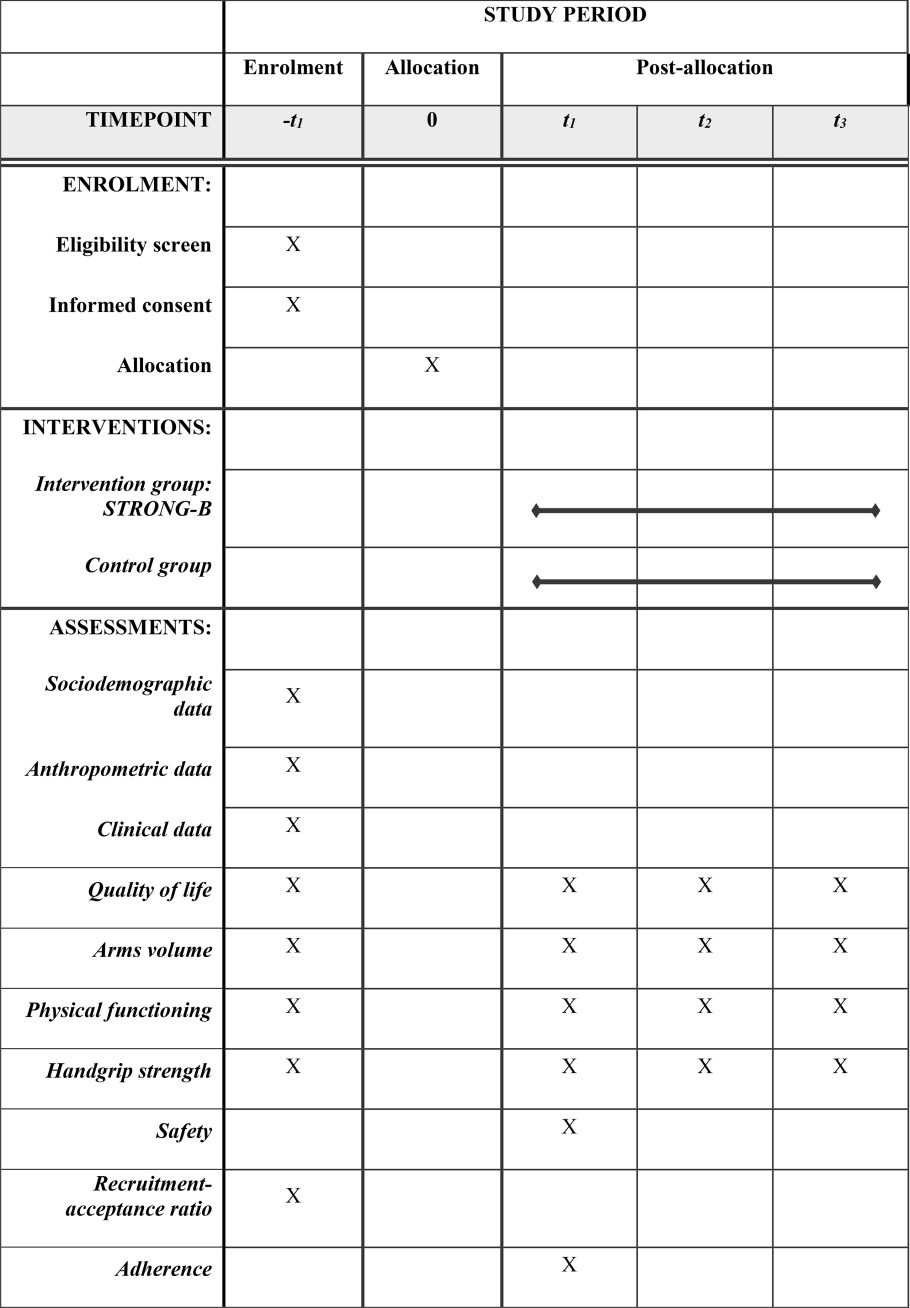
Schedule of enrolment, interventions, and assessments according to SPIRIT diagram.

**Figure 2:**
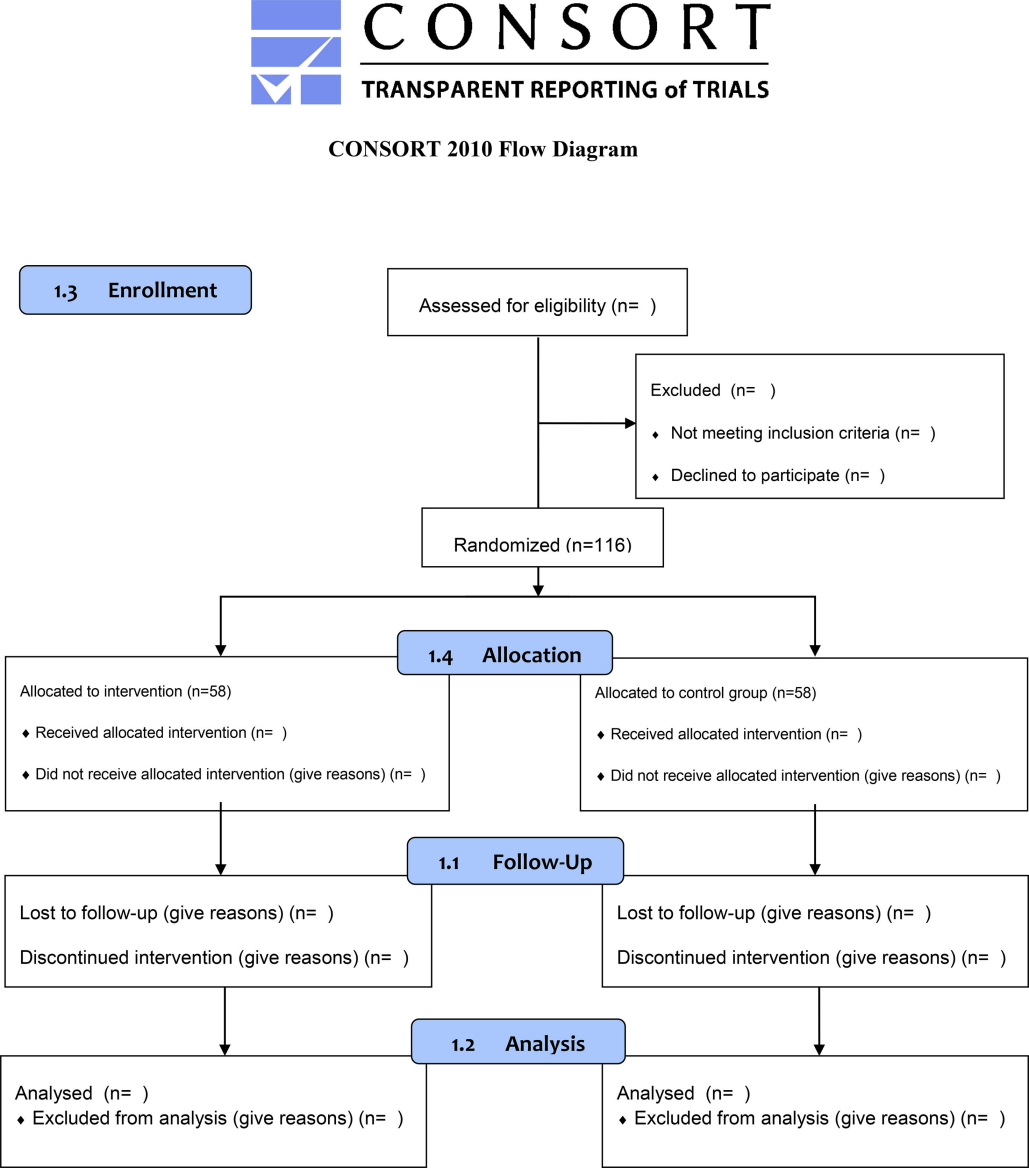
The proposed CONSORT diagram of enrolment, allocation, follow-up, and analysis through the study for each arm.

This manuscript is a study protocol for a two-arm, randomized controlled trial (STRONG-B). The protocol adheres to the Recommendations for Interventional Trials (SPIRIT) guidelines (44) and the CONSORT statement (45) (**Figures 1**, **2**, respectively). The STRONG-B trial has been registered in Clinicaltrial.gov (code NCT04821609).

The study population will be participants with breast cancer recruited from Complejo Asistencial Dr. Sótero Del Río by medical referral. Recruitment will take place in two distinct phases. First, a nurse will identify potential participants, and the health and medical records of volunteers will be further analyzed in a multidisciplinary committee, including medical oncologists, surgeons, and radio-oncologists. In the second phase, each volunteer’s eligibility will be confirmed by an oncologist during medical consultation. The same nurse of the first recruitment phase will then explain to each potential volunteer the purpose of the study and perform the informed consent process. Reasons for withdrawal from the study will be recorded.

### Eligibility Criteria

The following inclusion criteria will be applied: [a] women between 18 and 70 years old; [b] diagnosis of primary breast carcinoma with histological confirmation; [c] total or partial mastectomy with axillary node dissection; [d] sentinel node biopsy with positive axillary web syndrome (defined as cord in the subcutaneous tissue from the axilla into the ipsilateral upper arm); [e] sentinel node biopsy along with a body mass index between 30.0 and 39.9 kg/m^2^; [f] indication of adjuvant chemotherapy; and [g] participants able to understand and respond to simple instructions. The exclusion criteria will be: [a] >200 ml of difference in volume between arms; [b] antineoplastic treatment (chemotherapy, radiation therapy, or hormone therapy) before the current medical diagnosis; [c] stage IV breast cancer; [d] medical contraindication for exercise; [e] self-reported physical activity equivalent to the recent American College of Sports Medicine Physical Exercise Recommendation Guidelines for Patients and Cancer Survivors: 150 min/week of moderate aerobic exercise, or 75 min/week of vigorous aerobic exercise, and resistance training exercises at least two days per week; [f] body mass index <18.5 kg/m^2^ (indicative of malnutrition) or >40 kg/m^2^ (indicative of high cardiovascular risk); and [g] pregnancy.

### Intervention

STRONG-B is an exercise training intervention specifically developed for patients with breast cancer (46), which follows the guidelines of the American College of Sports Medicine with the specification for the frequency, intensity, time, and type of exercise prescription (31).

The training sessions include ten moderate-intensity resistance exercises for the upper (shoulder press, chest press, lateral pulldown, biceps curls, triceps extension) and lower limbs (squat, calf raise, leg press, leg extension, and leg curl). Each session lasts ~40 minutes and will be conducted twice a week for 12 consecutive weeks. Sessions will be supervised and guided by experienced physiotherapists, and conducted in groups of up to 3 participants.

The training will begin the week after the first chemotherapy session and will be conducted concomitantly with the regular treatment of each patient. Note that STRONG-B will never replace or interfere with the standard care. Each session will include a warm-up (5 minutes), followed by resistance training (30 minutes), and ending with a cooldown (5 minutes).

Resistance exercises will be performed at the maximum range of motion, using resistance machines or free weights as required. Rating of perceived exertion will be measured using a 0–10-point OMNI-Resistance Exercise Scale (minimal effort = 0; maximum effort = 10) to control perceived exercise intensity (47, 48).

During the first week, patients will perform two sets of 10 repetitions of each exercise either without resistance or with the lowest resistance available for a rating of perceived exertion of 2–4 (“easy” to “somewhat easy”). After this week, provided that no adverse events or symptoms are reported, resistance will be added so that each patient can perform three sets of 12 maximal repetitions (12-Repetition maximum) of each exercise with a rating of perceived exertion of 4-6 (“somewhat easy” to “somewhat hard”) (47, 48).

Progression will be supervised considering the patient’s symptoms (49). When patients can complete three consecutive sessions with the last volume and intensity, the load will be increased 5% to 10%. During the 12th week, the number of steps per day will be recorded using a smart bracelet [Huawei Band 4 (50)] as a surrogate of aerobic physical activity.

### Control Group

Patients in the control group will be referred to an early and prospective physical therapy program, as previously described (51). Therein, they will be evaluated and educated, but this program will not include the STRONG-B training. In the first session (before surgery), patients will receive oral and written education. Education will consist of care advice and eight exercises (**Supplementary Data**) to promote lymphatic and venous return and to maintain arm range of motion. We will recommend patients to perform these exercises in 3 sets of 10 repetitions, 2-3 times a day, for 3 months after surgery. Patients will be also educated to encourage aerobic physical activity. Physical exercise will be evaluated and supervised every three months after surgery, and educational information will be reminded. During the 12th week, the number of steps per day will be recorded using a smart bracelet [Huawei Band 4 (50)] as a surrogate of aerobic physical activity.

## Outcomes

### Main Outcome

Arm lymphedema will be assessed using a Perometer (NT 1000, Wuppertal), an optoelectrical imaging device that measures limb volume (52). The perometer is a valid and reliable tool [interclass correlation coefficient test-retest of 0.99 (52)]. The volume is expressed in milliliters and percentage relative to the contralateral arm. A difference in volume between arms of 200 mL or higher and a 10% difference between arms indicate lymphedema (12).

### Secondary Outcomes

Quality of life will be assessed using two questionnaires: [a] the European Organization for Research and Treatment of Cancer Quality of Life Questionnaire Core 30 v.3.0 [EORTC QLQ-C30; test/retest reliability between 0.82 and 0.91 (53)]; and [b] the European Organization for Research and Treatment of Breast Cancer-Specific Quality of Life Questionnaire BR23 [EORTC QLQ-BR23; Cronbach’s α between 0.46 and 0.94 (54)]. These questionnaires had been validated in the Spanish language and Chilean population (55).

Handgrip strength will be measured with a Hydraulic Hand Dynamometer (Jamar, United Kingdom). Patients will be asked to grip and squeeze the dynamometer as hard as possible. The maneuver will be conducted three times, with 1-min rest between attempts. Both hands (with surgery, without surgery) will be measured and compared. Results will be expressed in kilograms. Intra-instrument reliability and concurrent validity were tested using certified standard weights (r = 1.00), while inter-instrument reliability was good, between 0.80 and 0.83 (56). Further, there are reference values for a healthy Chilean population (57, 58).

Physical performance and mobility will be assessed with the 6-minute walk test (59). The test has been validated in patients with cancer and shows good reliability [intraclass correlation coefficient for test/retest was r=0.93 (60)]. Participants will be instructed to walk between two marks set 30 m apart as many times as possible over 6 min. The greater the distance covered, the greater mobility and general performance (59). Results will be expressed in meters walked.

### Sociodemographic and Clinical Data

Demographic data will include age, sex, and educational level. Anthropometric data will include weight, height, and body mass index. Clinical data will include disease stage, scheduled treatment, medical history, and current medication. Clinical data will be extracted from the patients’ electronic medical files by one study coordinator not blinded to patient allocation.

### Safety and Feasibility

The safety of the intervention will be assessed every week, tracking and monitoring adverse events according to the National Cancer Institute Common Terminology Criteria for Adverse Events version 5.0 (61). Researchers will meet weekly to review and discuss adverse events. All serious adverse events will be immediately reported to both ethics committees and will be reported in the results.

The number of patients who meet the eligibility criteria, the number of patients who agree or not participate, and the number of patients who withdraw from the study will be recorded and reported. Adherence will be calculated considering the number of patients who participated in all sessions and the number of patients who assisted to scheduled assessments.

### Sample Size

The sample size was determined using G*Power version 3.1.9.2, considering the arm volume as the primary outcome. A confidence level of 95%, statistical power of 90%, and a two-sided alpha of 5% were considered in the analysis. An effect size of 0.70 (d) has been chosen to detect differences in the arm volume in patients with BC undergoing chemotherapy; hence, 44 participants will be needed per group. Considering a dropout rate of 20%, 106 patients will be included in the study.

### Randomization

Patients will be randomly allocated (1:1 ratio) in two concealed numerical lists using the software Sealed Envelope™. The software generates different codes for each patient, which then identifies the group linked to the code. The code will be printed and placed in a dark closed envelope, which will be delivered to patients after baseline assessments. A blinded external researcher will perform the process. The allocation will be then reported to the nurse, who will subsequently inform each participant of the assigned group (intervention or control).

### Study Assessments

**Figure 3:**
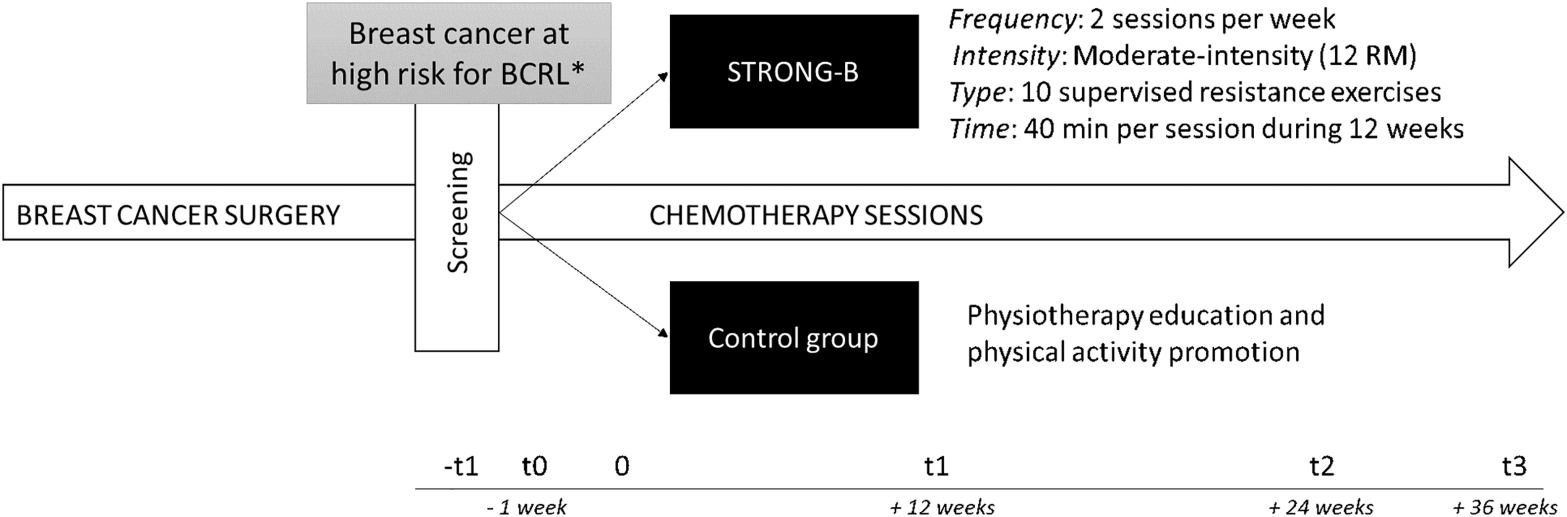
Study design. *Breast cancer at high risk for breast cancer-related lymphedema (BCRL) has been established as having had: [a] total or partial mastectomy with axillary node dissection; [2] sentinel node biopsy with positive axillary web syndrome; or [3] sentinel node biopsy along with a body mass index between 30.0 and 39.9; F, frequency; I, intensity; T, type; T, time.

**Figure 3** outlines the schedule for study outcome assessments. Assessments will be scheduled upfront, and patients will be regularly reminded *via* phone or email. Outcomes will be measured at four-time points: [t0] at baseline (up to 7 days before chemotherapy); [t1] after intervention (up to 5 days after the intervention ended); [t2] at three months of follow-up; and [t3] at six months of follow-up. Assessments will be made by an experienced researcher blinded to the allocation of patients.

### Data Management

Two researchers will manage data using predesigned data collection forms in Excel version 2016 (Microsoft Corporation, Redmond, WA, USA). Data will be regularly revised to ensure data quality. Patients will be identified by codes to ensure anonymity, and only the authors involved in the trial will have access to patients’ full identification details.

### Statistical Analyses

Analyses will be performed by a statistician blinded to the allocation of patients. Intention-to-treat and per-protocol approaches will be used. Normality assumption will be tested using the Shapiro-Wilk test. If the normality assumption is met, data will be presented using means and standard deviations. Otherwise, median and interquartile ranges will be reported. To assess the effect of the intervention, a multivariate linear regression model with repeated measurements will be adjusted. The model will include arm volume, quality of life, physical performance, and handgrip strength as response variables. Group (intervention, control), time (t0, t1, t2, t3), and the group×time interaction will be included as explanatory variables, adjusted for co-variables if required. The intervention’s effect will be also described by computing the effect size and 95% confidence limits. The following qualitative descriptors will be used: trivial <0.20, small 0.20-0.50, moderate 0.50-0.80, and large >0.80 (62). Significance will be set at p<0.05. Statistical analyses will be performed with STATA 15.1.

## Discussion

STRONG-B is a supervised resistance training intervention developed specifically for preventive BCRL in patients with breast cancer at high risk of BCRL. The training will be performed after breast cancer surgery and concomitantly with adjuvant chemotherapy. Considering the impact of cancer and the consequences of the treatments (6–14), the main objective will be to analyze the preventive effects of STRONG-B on arm volume, quality of life, physical performance, and handgrip strength. The feasibility, adherence, and security of the training will be also analyzed. STRONG-B is thus proposed as a tailored resistance training intervention supervised by experienced physiotherapists with multiple potential benefits for patients with breast cancer.

The economic burden of BCRL is high for patients and the health care system (28, 63–65). Three weeks of lymphatic decongestive treatment by a physiotherapist and follow-up require on average USD 2,648 per patient. Additionally, the required compression stockings cost on average USD 937 per patient (28). Finally, there are other non-medical costs such as transportation and loss of productivity due to morbidity and mortality. Together, these data highlight the relevance of developing strategies to prevent BCRL. Physical exercise may represent a plausible strategy (41, 66). If the STRONG-B training is successful, it would be easy to implement in usual care. Since resistance training is safe and well-tolerated in patients with breast cancer, the training may even be used as a primary prevention strategy against lymphedema and deterioration of quality of life.

The interplay and chronology between the factors leading to lymphedema are not well understood (67). Inflammation, adipose tissue remodeling, skin fibrosis, and deterioration of lymphatic vessels are known to be involved (67). Endolymphatic pressure increases immediately after a node resection, leading to irreversible histological changes in collecting lymphatic vessels (68). This process occurs even before the onset of BCRL and involves modifications in endothelial cells and basal membranes and the proliferation of collagen fibers. Interventions at these early stages seem thus essential. Notably, several studies have shown that lymph flow and shear stress are required for valve maintenance (69). Physical exercise can be used for that purpose. Complementing exercises with methods that detect small changes in arm volume [e.g. perometer and three-dimensional laser scanning (52, 70, 71)] will be helpful.

In addition, STRONG-B may have beneficial effects beyond BCRL in women with breast cancer. Long-lasting and high physical activity levels during cancer treatments have been shown to increase chemotherapy completion rate and reduce adverse effects such as fatigue, cardiotoxicity, and cognitive impairments (31, 72–74). This highlights the relevance and impact of chronic exercise interventions in individuals with cancer.

The protocol has certain limitations though. First, aerobic training will not be included, but aerobic physical activity will be encouraged and measured by bracelets in both groups. Second, implementing the training in small groups of patients may delay recruitment but seems safer in the current context of the COVID-19 pandemic. Implementing rehabilitation through telemedicine may also be useful and complement interventions in the future (75, 76). Third, patients undergoing chemotherapy may feel fatigue or discomfort, thus reducing adherence (77); to prevent this issue, STRONG-B was designed considering the preferences of breast cancer survivors (46). Finally, due to the nature of the intervention, neither participants nor health care workers will be blinded to the group assigned to each participant (78).

In conclusion, the STRONG-B training is proposed as a tailored supervised resistance training for patients with breast cancer at high risk for BCRL. This study will attempt to prevent or mitigate BCRL in a population that, due to both intrinsic and extrinsic factors, will commonly suffer from BCRL

## Ethics Statement

The study was approved by the “Comité Ético del Servicio de Salud Metropolitano Sur Oriente” (September 24th, 2020) and the “Comité Ético Científico Ciencias de la Salud UC (200310003, 8 October 2020). All patients will receive written and verbal information before starting the study, and written and oral informed consent will be obtained from all participants in the study.

## Author Contributions

Conceptualization: KR-P, CS-R, MP-G, and IC-V. Formal analysis: KR-P, ML-G, and IC-V. Methodology: RF-V and AR-P. Project administration: MA-B. Supervision: CS-R, GM-P, and IC-V. Writing – original draft: KR-P, ML-G, and IC-V. Writing – review & editing: KR-P, ML-G, CS-R, MP-G, MA-B, RF-V, AR-P, GM-P, and IC-V. All authors contributed to the article and approved the submitted version.

## Funding

The study is funded by ANID+FONDEF/XVII Concurso Nacional de Proyectos de Investigación y Desarrollo en Salud, Fonis (SA20I0060).

## Conflict of Interest

The authors declare that the research was conducted in the absence of any commercial or financial relationships that could be construed as a potential conflict of interest.

## Publisher’s Note

All claims expressed in this article are solely those of the authors and do not necessarily represent those of their affiliated organizations, or those of the publisher, the editors and the reviewers. Any product that may be evaluated in this article, or claim that may be made by its manufacturer, is not guaranteed or endorsed by the publisher.
