# Supplementary material for: Effect of Supervised Resistance Training on Arm Volume, Quality of Life and Physical Perfomance Among Women at High Risk for Breast Cancer-Related Lymphedema: A Study Protocol for a Randomized Controlled Trial (STRONG-B)
Source: Front Oncol. 2022 Mar 1;12:850564. doi: 10.3389/fonc.2022.850564 (PMC8921986; doi:10.3389/fonc.2022.850564)

Supplementary Data. Exercises recommended to the control group.

1. Active assisted shoulder flexion with stick. Lift the arms with a stick above the head. Keep the elbows as straight as possible. Maintain the elevation 5-10 seconds, then slowly lower the arms.

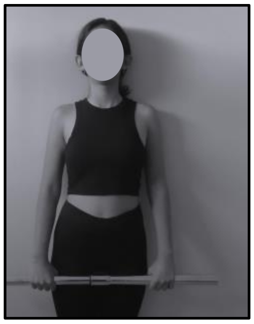
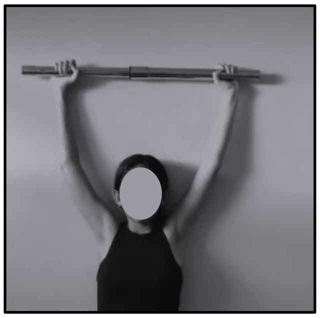

2. Shoulder abduction with external rotation. Clasp the hands behind the neck with the elbows pointing toward the front. Move the elbows toward the wall, and maintain for 5-10 seconds.

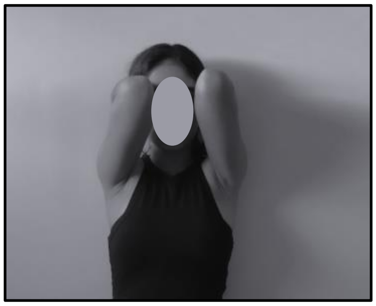
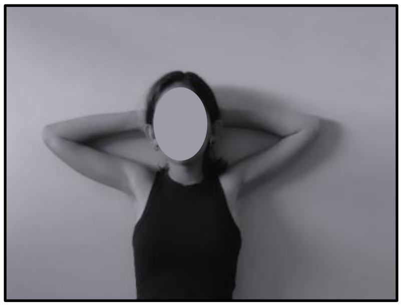

3. Shoulder external rotation with scapular depression. Raise and lower the stick behind the head. Hold the stick with both hands above head, palms facing forward. Lower de stick until it is resting behind the neck. Maintain the position for 5-10 seconds.

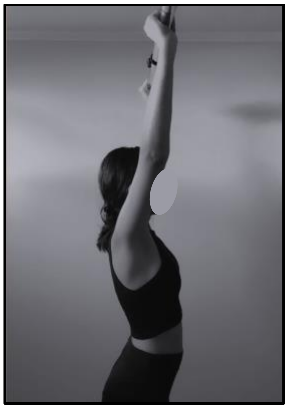
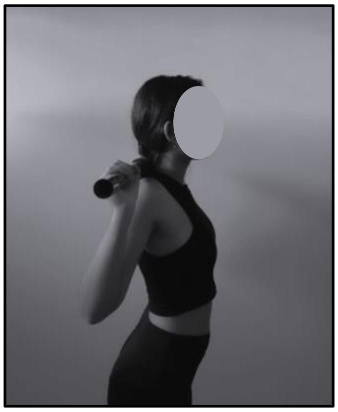

4. Scapular retraction. Squeeze the shoulder blades together, bringing elbows toward your spine. Return to the starting position.

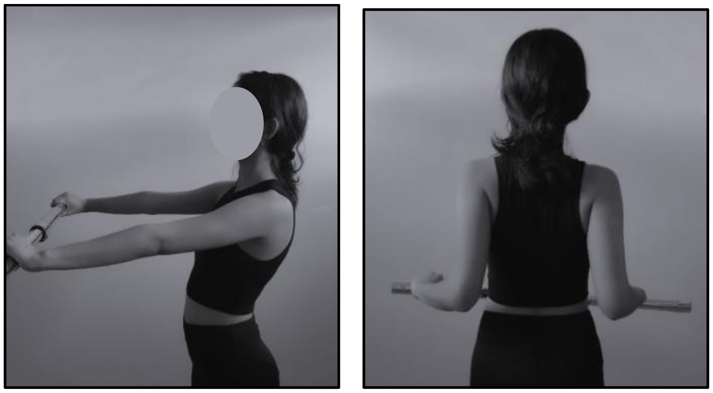

5. Shoulder rotation with a stick. Grasp the stick with one hand and cup the other end of the stick with the other hand. Keep the elbow of the shoulder being stretched against the side of the body and push the stick horizontally to feel a slight pull without pain. Hold for 5 seconds and then relax.

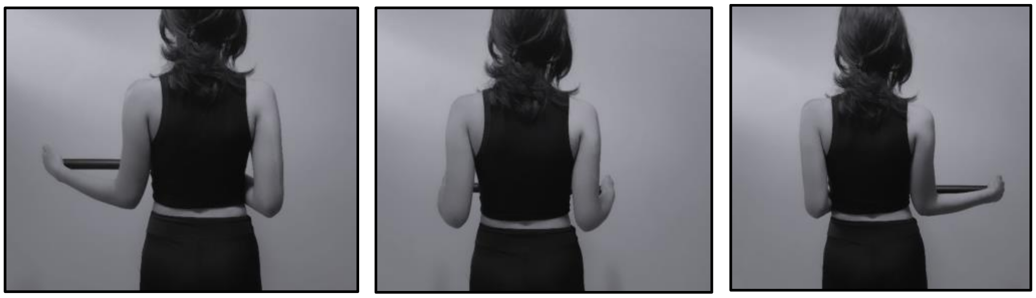

6. Start with the fingers out straight. Make a fist, ensuring each finger joint is bending as much as possible. Straighten the fingers as much as possible.

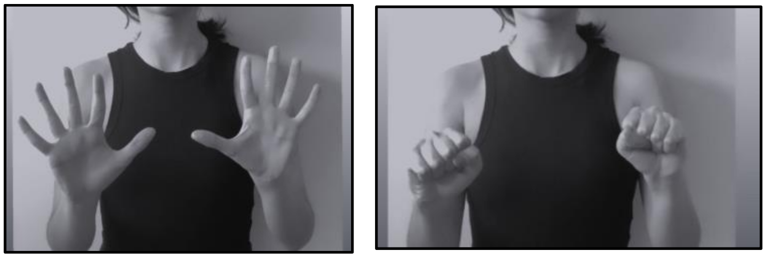

7. Spread the fingers wide apart, and then bring them together.

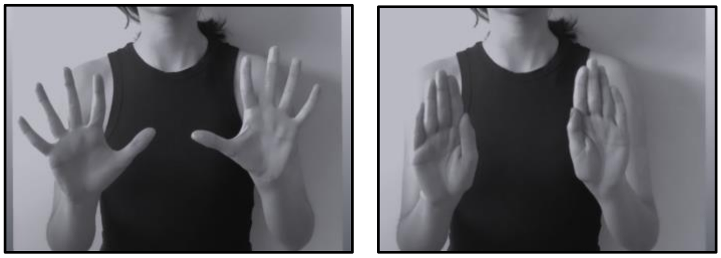

8. With the wrists and fingers straight, bend the fingers at the knuckles.

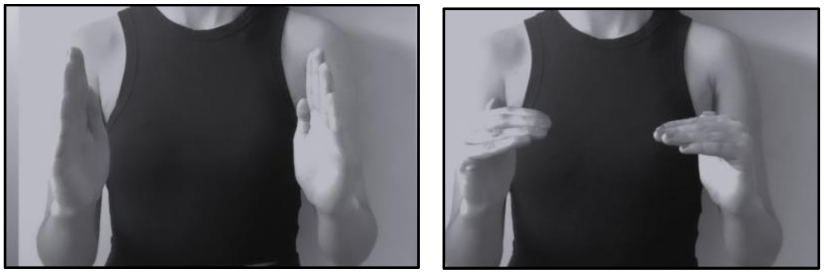

Supplement: Supplementary file 1 [file DataSheet_1.pdf]
